# Radiative and Magnetically Stimulated Evolution of Nanostructured Complexes in Silicon Surface Layers

**DOI:** 10.3390/ma15124052

**Published:** 2022-06-07

**Authors:** Dmytro Slobodzyan, Markiyan Kushlyk, Roman Lys, Josyp Shykorjak, Andriy Luchechko, Marta Żyłka, Wojciech Żyłka, Yaroslav Shpotyuk, Bohdan Pavlyk

**Affiliations:** 1 Deptartment of Sensor and Semiconductor Electronics, Ivan Franko National University of Lviv, 107, Tarnavskoho Str., 79017 Lviv, Ukraine; markiyan.kushlyk@lnu.edu.ua (M.K.); roman.lys@lnu.edu.ua (R.L.); yosyp.shykoryak@lnu.edu.ua (J.S.); andriy.luchechko@lnu.edu.ua (A.L.); bohdan.pavlyk@lnu.edu.ua (B.P.); 2 Department of Aerospace Engineering, Rzeszow University of Technology, 8, Powstańców Warszawy Av., 35-959 Rzeszow, Poland; mzylka@prz.edu.pl; 3 Institute of Materials Engineering, University of Rzeszow, 1, Pigonia Str., 35-310 Rzeszow, Poland; wzylka@ur.edu.pl; 4 Institute of Physics, University of Rzeszow, 1, Pigonia Str., 35-310 Rzeszow, Poland

**Keywords:** silicon, nanostructure complexes, magnetic field, X-irradiation, current–voltage characteristic, capacitance–voltage characteristic, IR spectra, deep level

## Abstract

The effect of a weak magnetic field (B = 0.17 T) and X-irradiation (D < 520 Gy) on the rearrangement of the defective structure of near-surface p-type silicon layers was studied. It was established that the effect of these external fields increases the positive accumulated charge in the region of spatial charge (RSC) and in the SiO_2_ dielectric layer. This can be caused by both defects in the near-surface layer of the semiconductor and impurities contained in the dielectric layer, which can generate charge carriers. It was found that the near-surface layers of the barrier structures contain only one deep level in the silicon band gap, with an activation energy of Ev + 0.38 eV. This energy level corresponds to a complex of silicon interstitial atoms Si_I_+Si_I_. When X-irradiated with a dose of 520 Gy, a new level with the energy of Ev + 0.45 eV was observed. This level corresponds to a point boron radiation defect in the interstitial site (B_I_). These two types of defect are effective in obtaining charge carriers, and cause deterioration of the rectifier properties of the silicon barrier structures. It was established that the silicon surface is quite active, and adsorbs organic atoms and molecules from the atmosphere, forming bonds. It was shown that the effect of a magnetic field causes the decay of adsorbed complexes at the Si–SiO_2_ interface. The released hydrogen is captured by acceptor levels and, as a result, the concentration of more complex Si–H_3_ complexes increases that of O_3_–Si–H.

## 1. Introduction

Today, silicon is an important material for the manufacture of electronic devices for various purposes. Therefore, a significant number of scientific publications devoted to the study of its properties have accumulated over the past few decades. However, the regular miniaturization of the elements stimulates an increase in the level of requirement for the perfection of the internal structure of silicon, as well as for the reliability of devices based on it [1,2,3]. This, in turn, sets a high priority for the study of silicon nanoscale complexes [4]—especially Si clusters [5,6,7,8]—to improve the properties of nanostructured materials.

It has already been established that purposeful control of the composition of a silicon cluster containing organic elements (e.g., C, O, H) makes it possible to obtain optimal optical parameters of light-emitting and light-absorbing structures with nanostructured objects [9]. Clusters of silicon atoms doped with transition metals as a basis for the formation of stable clusters with specified electrical and optical properties are promising materials for nanoelectronics [10,11]. This study raises the question of the stability of a certain type of silicon-containing cluster with respect to their components and atomic configuration.

The formation of clusters with given parameters in the near-surface layers of the semiconductor is also an important task of electronic technology. Vacuum-plasma methods are mainly used for this purpose. In particular, vacuum deposition of metal atoms on a silicon surface saturated with restrictive molecules was used in [12]. Another method is the direct implantation of carbon and hydrogen with subsequent temperature annealing [13]. As a result, it is possible to obtain improvements in the characteristics of silicon structures [13,14,15]. However, in the presence of a significant number of linear defects, such techniques can cause mechanical damage to the surface [16].

However, technological operations of the manufacturing of electronic devices [17,18,19], operating under extreme external fields [20,21,22,23,24], can stimulate the creation of defect complexes based on point defects (e.g., vacancies and interstitial silicon atoms) [25,26,27] and their further interaction and clustering [28,29].

Small external factors can also stimulate the restructuring processes of existing nanoscale complexes and their clusters in semiconductor structures. This is mainly due to the excitation of the electronic subsystems of defects that exist on the silicon surface and at the Si–SiO_2_ boundary [30,31,32,33,34,35,36]. In particular, small doses of ionizing radiation can cause the generation of non-equilibrium electrons and holes, which can be captured by defective complexes [30,31,32]. A weak magnetic field can stimulate the reorientation of the electron spin of the atomic defects [33,34,35,36]. Consequently, new nanoscale complexes are formed, which have other kinetic parameters and change the characteristics of the silicon structures [37,38,39,40]. Therefore, the influence of such fields can be used as an additional technological operation to improve the characteristics of silicon structures.

Thus, the raw material purification, modification of silicon synthesis methods with controlled defect content, and formation of electronic structures with an enhanced content of nanostructured complexes in the near-surface layers remain relevant in the transition from micro- to nanoelectronics today.

This study aimed to investigate the effects of small doses of X-rays and a weak magnetic field on the reconstruction of the defective subsystems of the surface and near-surface layers of p-Si with a low content of growth defects, as well as to establish the reasons for the corresponding changes in the characteristics of diode structures created based on this semiconductor.

## 2. Materials and Methods

Boron-doped p-type Cz-Si with resistivity of ρ = 10 ohm·cm was used for the investigations in this work. The synthesis of the monocrystalline sample was performed by the «ROST» company (Kyiv, Ukraine). Samples were cut from a single crystal silicon disk with a flat surface orientation (111). The dimensions of the experimental samples were 0.5 × 3 × 8 mm^3^. Chemical etching was used to reduce the thickness of the natural SiO_2_ layer (between the metal and the semiconductor) and prepare the surface for the formation of metal contacts. It should be noted that this SiO_2_ dielectric layer was not purposefully grown, as compared to MOSFET technology [13,19,22,30]. It still exists even after the chemical etching of the silicon samples in HF+HNO_3_ acid solution. The thickness of silicon oxide can reach several nanometers by using this technological operation. 

**Figure 1:**
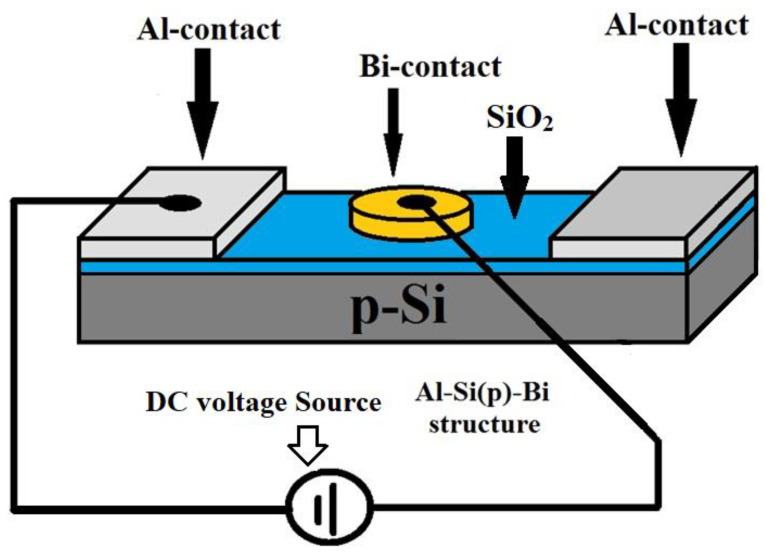
Bi-Si-Al surface barrier structure.

The objective of this work was to form a surface barrier structure (SBS) of Bi-Si-Al (Figure 1), and to investigate the changes in their electrophysical characteristics under the influence of X-radiation (below 520 Gy) and magnetic field (0.17 T).

The thickness of the Al and Bi contacts was 0.5 μm. The metal thickness was controlled by a quartz oscillator during the vacuum deposition on a silicon substrate.

Irradiation of the samples was carried out with an X-ray tube (V = 45 kV, I = 8 mA, W-anode). The magnetic field was applied with different durations and a constant induction of 0.17 T.

The influence of external fields with similar parameters has been studied and analyzed in a previous work [41]. However, the investigated material was silicon, with a high concentration of surface defects (10^2^ cm^−2^).

The electrophysical properties of SBS were studied by investigating the current–voltage characteristics (IVCs) and high-frequency capacitance–voltage characteristics (CVCs). From experimental C–V characteristics, the distribution of surface-state density in silicon’s band gap on the boundary of Si–SiO_2_ was calculated [42]. Defect formation processes on the surface and subsurface layers of silicon crystals were analyzed by IR spectroscopy and deep-level capacitance-modulation spectroscopy (DLCMS) [9].

Transmission spectra were recorded using a Shimadzu UV-3600 spectrometer in the 1000–3000 nm wavelength range. The absorption spectra were recorded using a Specord M20 IR spectrometer in the wavelength range of 2.5–25 microns. Deep-level spectra were recorded with an HU7-1 capacitive spectrometer in the 77–400 K temperature range. Measuring the parameters of deep levels in this temperature range enables study of the radiation-stimulated rearrangement of defects without the additional influence of the temperature field as a stimulating factor.

Chemical removal of the metal films was performed after the electrophysical characteristics of the silicon structures were measured. Next, selective surface etching and structural studies of silicon morphology were performed with an LUMAM I-3 optical microscope and a Solver P47-PRO atomic-force microscope.

## 3. Results and Discussion

**Figure 2:**
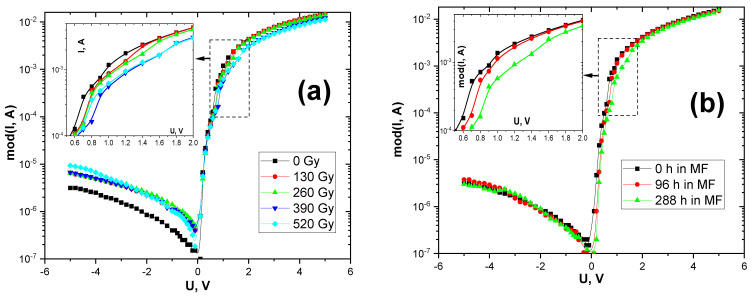
Radiative (**a**) and magnetic (**b**) stimulated changes of current–voltage characteristics in the Bi-Si-Al surface barrier structures.

Figure 2a shows the changes in the current–voltage characteristics of the SBS under the influence of small doses of X-irradiation. It can be seen that the value of the reverse current increased approximately twofold even at the beginning of the irradiation (130 Gy). The steepness of the straight section of the current–voltage characteristics also changed during irradiation. In particular, at forward voltages greater than 0.6 V, the current value decreased by 20% every 260 Gy (inset in Figure 2a).

Figure 2b shows the change in the current–voltage characteristics of the SBS after exposure to a magnetic field for 4 and 12 days. It can be seen that the value of the forward current decreased with increasing exposure time under the magnetic field. However, the form of dependence of the reverse current on the voltage did not change under the effect of the magnetic field.

**Figure 3:**
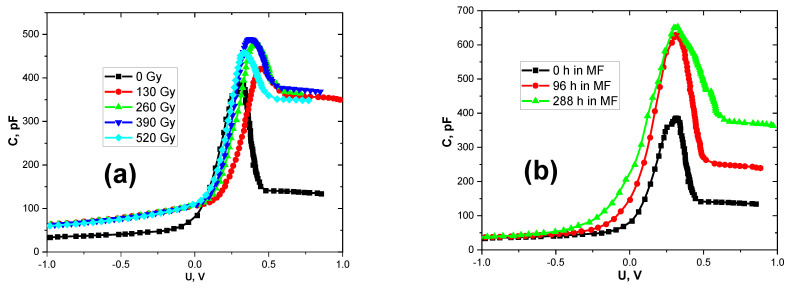
Radiative (**a**) and magnetic (**b**) stimulated changes of capacitance–voltage characteristics in the Bi-Si-Al surface barrier structures.

More information about the changes in current–voltage characteristics under the influence of radiation and magnetic field can be obtained by the analysis of capacitance–voltage characteristics (Figure 3). Both external fields led to the following changes in the CVCs of the barrier structures: (1) the value of the maximum CVC changed; (2) the capacitance values of the structures changed in the region of positive voltages (more than 0.5 V); (3) the nature of the capacitance in the region of negative voltages changed. 

As shown in [41], the maximum CVC corresponds to the accumulated positive charge in the SiO_2_. The presence of this oxide layer between the metal and the semiconductor could not be completely removed during the preparation of the barrier structures. The charge increase was observed both under the effect of radiation and under the effect of a magnetic field. However, at absorbed doses less than 390 Gy, the maximum capacitance increased by 25%, and then began to decrease. The magnetic field contributed to the accumulation of charge in the SiO_2_ dielectric layer. Moreover, the capacitance increased approximately 1.8-fold relative to the initial value.

Similarly, the capacitance value of the structures changed in the region of positive voltages. This parameter corresponds to the value of the spatial charge region (SCR) [41]. When the charge is accumulated in the SCR, the potential barrier value increases. This causes a decrease in the forward current through the Bi-Si(p) contact. It should be noted that this correlates well with the results shown in Figure 2.

Although the used fields were different in physical nature, there were similar results of their influence on the electrophysical characteristics of the silicon-based barrier structures. Specifically, there were processes associated with the accumulation of charge in the SCR and the dielectric layer, both under the effect of radiation and under the effect of a magnetic field. This could be caused by defects in the near-surface layer of the semiconductor or impurities contained in the dielectric layer, which can generate charge carriers.

The general picture of changes in the charge state of the surface is given by calculating the density distribution of surface states (SS) N_SS_ = f(E) at the dielectric–semiconductor interface. The general approach in these calculations is based on comparing the ideal metal–dielectric–semiconductor structures calculated by CVC, with experimental dependences of capacitance and differential conductivity on the bias voltage.

It is assumed that the surface states change the appearance of the equilibrium C–V characteristics, since part of the charge induced by the external field is captured in these states. As a result, the field in the semiconductor is shielded, and in the dielectric layer it increases. Thus, it is necessary to apply an additional displacement ΔUg in order to compensate for the field in the dielectric layer and obtain the desired curvature of the energy zones in the semiconductor [43].

**Figure 4:**
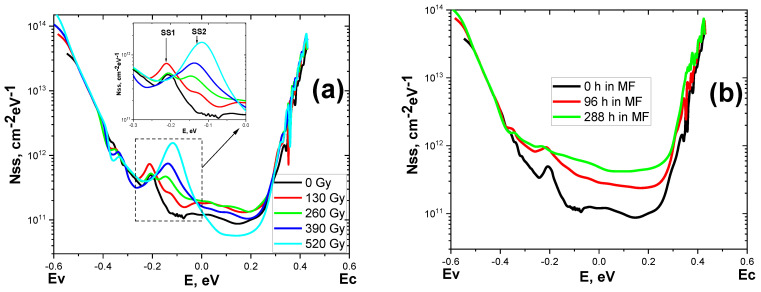
Radiative (**a**) and magnetically (**b**) stimulated changes in the charge states of the Si–SiO_2_ interface in the Bi-Si-Al surface barrier structures (distribution of the density of fast surface states in the silicon band gap).

From the previously presented radiation- and magnetostimulated CVCs (Figure 3) the density of surface states in the band gap (BG) of silicon was mathematically calculated (Figure 4). The zero point on the energy scale corresponds to the middle of the BG. The states of the lower half of the BG are placed on the left, and the upper half is placed on the right. For the original sample (black curve in Figure 4a,b), a continuous spectrum of states is characteristic [44]. 

Moreover, the concentration of levels in the lower part of the SS was higher than in the upper part. This was due to the presence of an alloying acceptor of boron doping. However, near the middle of the BG, a maximum (SS1) with the energy of Ev + 0.39 eV was observed. This maximum may correspond to an electrically active point defect.

The initial irradiation (130 Gy) of the sample led to a slight increase in the density of the states located near the middle of the BG (Figure 4a). A dose of 260 Gy caused a decrease in the value of SS1 and the appearance of a new SS2 maximum in the spectrum. This peak, with an energy of Ev + 0.49 eV, was closer to the middle of the BG than SS1, and may correspond to another type of point defect. As the absorbed dose increased, the amplitude of SS1 decreased, while that of SS2 increased. Consequently, there was a process of rearrangement of point defects in the near-surface silicon layer at the Si–SiO_2_ interface during radiation exposure.

Figure 4b shows magnetically stimulated changes in the density of states. It is worth noting that 4 and 12 days of exposure to the SBS in the magnetic field stimulated an intensive increase in the concentration of surface states over almost the entire width of the BG. Moreover, the SS1 maximum could no longer be allocated. 

**Figure 5:**
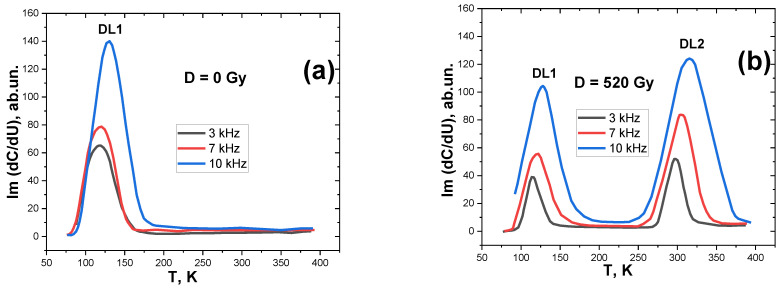
Capacitive-modulation spectrum of deep levels in the band gap of the Bi-Si-Al surface barrier structures before (**a**) and after (**b**) X-irradiation with dose D = 520 Gy.

**Figure 6:**
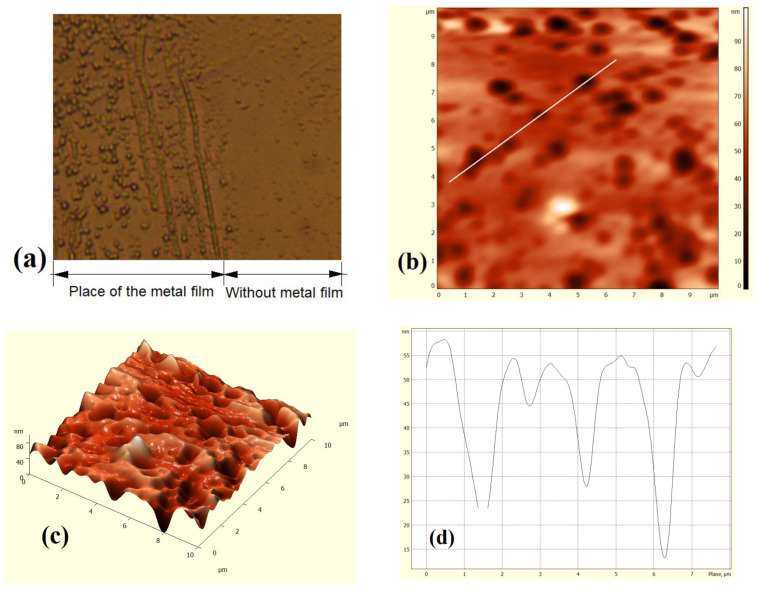
Structural studies of the silicon (111) surface: microphotography of the silicon surface taken with an LUMAM I-3 optical microscope (x 150) (**a**); silicon morphology (from the side where the covered metal film was previously) obtained using a Solver P47-PRO atomic-force microscope (**b**–**d**).

**Figure 7:**
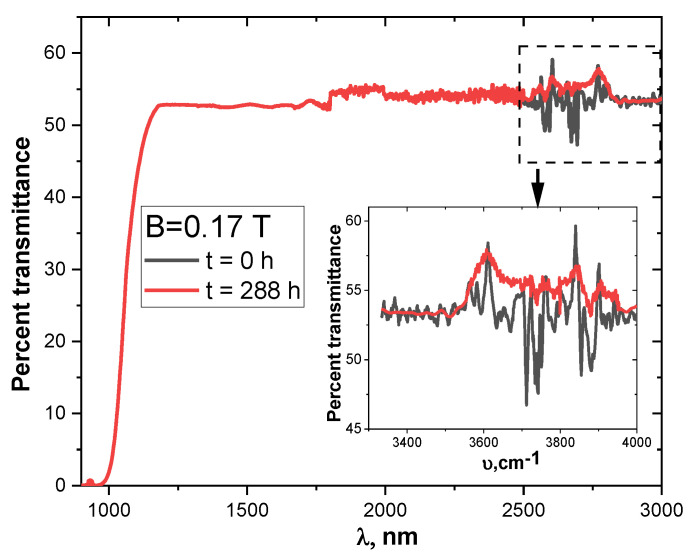
Infrared transmittance spectra of the initial p-Si samples before and after 288 h of exposure to the magnetic field.

To determine the cause of the changes, a study of the rearrangement of electrically active defects by the DLCMS method (Figure 5) and of adsorbed complexes by IR spectroscopy (Figure 6 and Figure 7) in the near-surface layers of silicon was carried out. 

The temperature dependence of the imaginary component of the capacitance on the modulating voltage of the studied barrier structures is presented in Figure 5. According to this method, the activation energy of deep levels (DLs) in the BG of a semiconductor was determined [9]. As can be seen, before irradiation (Figure 5a), the near-surface layers of the barrier structures contained only one deep level (DL1), with an activation energy of Ev + 0.38 eV. This correlates well with the presented state density spectrum (Figure 4).

Establishing conformity between the energy level in the BG and a certain type of defect is complicated. The depth of the energy level lies between the values corresponding to the K-center (Ev + 0.35 eV, Ev + 0.38 eV) and the interstitial silicon (Si_I_) atom (Ev + 0.40 eV). It should be noted that the K-center is a complex of an interstitial oxygen atom and an interstitial carbon atom. K-centers can determine the lifetime of non-equilibrium carriers in p-type-conductivity silicon [45]. However, this defect is related to radiative creation [46], and its presence in the original sample of silicon is questionable. 

We can assume that the level Ev + 0.38 eV corresponds to a complex of silicon interstitial atoms Si_I_+Si_I_ [9,45,47]. Such a defect is not growth-related, since silicon is synthesized for microelectronics, and this requires additional purification methods of raw materials when synthesizing substrates. Therefore, the technological conditions for manufacturing the barrier structures could cause the formation of this defect. 

In particular, surface preparation for making contacts (e.g., grinding, polishing, annealing) and direct deposition of metal to the surface creates additional mechanical stresses in the near-surface layer, and increases the rate of defect diffusion [48]. This was confirmed by microscopic studies of the silicon surface (Figure 6). 

Figure 6a shows the image of the silicon surface (111) covered with a metal film. It can be seen that an increased concentration of structural inhomogeneity is formed under the sputtered film on the crystal’s surface. Analysis of Figure 6b,c shows that this inhomogeneity constitutes a group of structural surface defects that differ from the dislocation in terms of the depth and shape of their edges (Figure 6d). According to [48], the authors consider these defects to be an aggregation of point defects.

Conversely, for the surface on which the film was not deposited, an increase in surface defect concentration was not observed. This allows us to claim the heterogeneous properties of the silicon metal structure.

As a result, the concentration of defects in this layer will be much higher compared to the volume. 

Together with the oxygen and hydrogen atoms that are present in the dielectric layer, they can form electrically active complexes [31].

In the initial stages of irradiation (D < 390 Gy) at the Si–SiO_2_ interface, the process of electron–hole pair generation and localization of holes in the dielectric layer takes place [30,49], and the charge state of the surface changes. However, according to previous research [41], the diffusion coefficient of intrinsic interstitial silicon atoms increases at low doses of irradiation.

A further increase in the amount of absorbed dose causes an increase in the concentration of interstitial silicon atoms in the near-surface layer, as well as changes in its charge state.

When structure is irradiated with a dose of 520 Gy, there is a decrease in the amplitude of DL1 and the appearance of a new level—DL2—with the energy of Ev + 0.45 eV. This level corresponds to a boron point defect in the interstitial sites (B_I_). This is thought to be one of the radiation defects in p-type silicon that is observed under high-energy irradiation [45,46]. 

However, this process is also possible via X-irradiation, whereby interstitial silicon can push a boron atom into the interstitial site. An electrically active defect captures electrons and holes generated by radiation, and changes the charge state. This causes the migration of interstitial silicon from the tetrahedral position to the hexagonal position and conversely, and then the displacement of boron into the interstitial site [45,47]. 

This causes changes in the energy spectrum of the BG of silicon. Specifically, a new defect B_I_ is formed, with the corresponding deep level (DL2):

(1) $$B_{S}+{\mathrm{Si}}_{I}\to B_{I}$$

However, if we consider that the displacement of boron at the interstitial site is accompanied by a loss in its electrical activity, the left part’s expression (1) should consist of components with opposite charge states or components with no electrical activity. However, the interstitial silicon in p-Si is characterized by the charge states Si_I_^+^ or Si_I_^++^, and in the initial stages of irradiation (130–260 Gy) some of the boron atoms can transition to an electrically active state [45,46,47]. Thus, the following chemical reaction takes place:

(2) $$B_{S}^{-}+{\mathrm{Si}}_{I}^{+}\left({\mathrm{Si}}_{I}^{++}\right)\to B_{I}$$

The analysis of the results of DL capacitive spectroscopy is in good agreement with the analysis of the density of the surface states. These two types of defect are effective in obtaining charge carriers, and cause deterioration of the rectifier properties of the silicon barrier structures.

The IR transmission spectra studied in the range from 1 to 3 µm of a silicon sample before and after magnetic processing are presented in Figure 7. In the near-IR region, there was no effect of the magnetic field on the spectrum. However, the transmission oscillations were in the 3000–4000 cm^−1^ range, showing the existence of absorption centers on the silicon surface (inset in Figure 7). After 288 h of exposure to a magnetic field, the transmittance value in this region increased, indicating the neutralization of such complexes.

Previous research showed that a weak magnetic field can cause the decay of adsorbed complexes at the Si–SiO_2_ interface [33,34,35,41]. This was confirmed by the analysis of IR spectra in the 500 to 4000 cm^−1^ range. 

**Figure 8:**
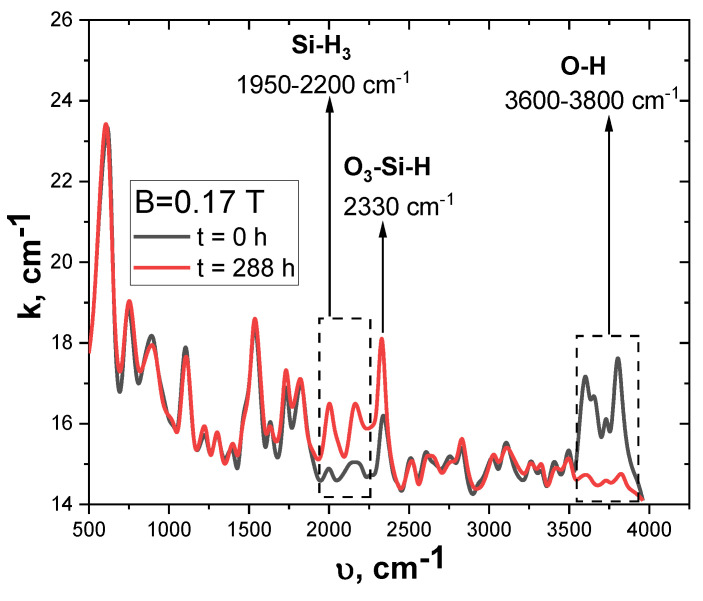
Infrared absorption spectra of the initial p-Si samples before and after 288 h of exposure to the magnetic field.

Figure 8 shows the IR absorption spectrum of silicon before and after magnetic processing. The silicon surface is quite active, and adsorbs organic atoms and molecules from the atmosphere, forming bonds [9]. As a result of the effect of the magnetic field, the amplitude of the absorption coefficient decreases in the range of 3500–4000 cm^−1^ and grows in the range from 1950 to 2350 cm^−1^. 

The maxima of the IR spectrum in the frequency range from 3000 cm^−1^ to 4000 cm^−1^ are responsible for the absorption of vibrations of O–H bonds in water molecules and in Si–O–H bonds [50]. 

Under the effect of the magnetic field, these complexes decay; consequently, the released hydrogen is captured by acceptor levels, and the concentration of more complicated Si–H_3_ (1950–2200 cm^−1^) and O_3_–Si–H (2330 cm^−1^) complexes increases. 

According to previous works [33,34,35], a spin-dependent process of chemical bonds breaking in nanoclusters of structural defects (e.g., Si-H, Si-OH) occurs under the effect of the magnetic field. Hydrogen ions formed after the decomposition of chemical bonds diffuse across the crystal and passivate the acceptor and donor bonds.

Moreover, the effect of the magnetic field can lead to dynamic polarization of the nuclei of silicon Si^29^ isotopes, as well as the polarization of the spins of silicon electrons and the spins of impurity (oxygen) electrons due to ultrafine interaction with the polarized nuclei [51]. A change in the spin orientation of an electron involved in the formation of strained Si–Si bonds and bonds in SiO_3_ oxide precipitates at the Si–SiO_2_ boundary leads to the filling of anti-binding orbitals and the decay of the chemical bonds. As a result, a P_b_ center and a silicon atom are formed. The silicon atom is bound to three oxygen atoms, and has one electron in a free orbital [52,53]. As a result, this complex can capture the hydrogen ion, forming the structure O_3_–Si–H [54]. This causes a decrease in direct current through the barrier (Figure 2b), the accumulation of charge in the SiO_2_ layer (Figure 3b), and an increase in the density of the surface states (Figure 4b).

## 4. Conclusions

The surface and near-surface layers of the semiconductor are enriched with nanoscale defects and their complexes under the fabrication of Bi-Si-Al surface barrier structures based on single-crystalline silicon. The criteria for this process are the initial defect state of the single crystal and the technological conditions for obtaining the structure. One type of electrically active center was detected in the near-surface layers of silicon under metal films using deep-level capacitance-modulation spectroscopy. The deep level with ionization energy E_V_ + 0.38 eV corresponds to complexes based on the interstitial silicon atoms (Si_I_+Si_I_). A significant number of surface complexes based on oxygen and hydrogen atoms were detected by IR spectroscopy.

Small doses of X-irradiation (D ~ 520 Gy) stimulate changes in the charge states of (Si_I_+Si_I_) defect complexes. As a result, the impurity atoms are pushed out from the lattice sites into the interstitial sites, and a new deep-level electrically active defect (B_I_) with ionization energy of E_V_ + 0.45 eV is formed. A weak constant magnetic field (B = 0.17 T) stimulates the rupture of chemical bonds in hydrogen- and oxygen-containing complexes on the silicon surface. This is due to the changes in the direction of the electron spins that form these bonds. As consequence, new surface centers (O_3_–Si–H and Si–H_3_) are formed, and acceptor bonds are passivated.

The presented results show the ability of X-rays and magnetic fields to modify the defective composition of the silicon surface and the near-surface layers of metal–Si, Si–SiO_2_, and metal–SiO_2_–Si structures. Further study of the weak fields’ effect on silicon structures—especially in combination with elastic–plastic deformation and temperature annealing in active atmospheres—may improve existing knowledge about targeted enrichment of the near-surface layers with nanoscale complexes with specified properties. The research on the correlation between the effects of the external fields (radiation, deformation, magnetic, etc.) and the changes in properties is remarkable for the understanding of the structural processes in silicon. In addition, it meets the need for silicon-based materials with targeted modification of their properties.

## Data Availability

The data presented in this study are available upon request from the corresponding author.
